# Supplementary material for: Time-series transcriptome analysis identified differentially expressed genes in broiler chicken infected with mixed Eimeria species
Source: Front Genet. 2022 Aug 8;13:886781. doi: 10.3389/fgene.2022.886781 (PMC9393255; doi:10.3389/fgene.2022.886781)
Supplement: Supplementary file 2 [file DataSheet1.ZIP › 4dpi_KEGG.Gsea.1625100298584/gsea_report_for_0_1625100298584.html]

Report for 0 1625100298584 [GSEA]

| GS  follow link to MSigDB | GS DETAILS | SIZE | ES | NES | NOM p-val | FDR q-val | FWER p-val | RANK AT MAX | LEADING EDGE || 1 | KEGG\_RIBOSOME | Details ... | 72 | -0.79 | -3.11 | 0.000 | 0.000 | 0.000 | 1964 | tags=86%, list=16%, signal=102% |
| 2 | KEGG\_NITROGEN\_METABOLISM | Details ... | 16 | -0.64 | -1.79 | 0.003 | 0.107 | 0.199 | 142 | tags=25%, list=1%, signal=25% |
| 3 | KEGG\_OXIDATIVE\_PHOSPHORYLATION | Details ... | 82 | -0.43 | -1.77 | 0.002 | 0.090 | 0.244 | 4262 | tags=51%, list=35%, signal=79% |
| 4 | KEGG\_LYSOSOME | Details ... | 103 | -0.41 | -1.74 | 0.000 | 0.087 | 0.302 | 3447 | tags=50%, list=29%, signal=70% |
| 5 | KEGG\_PORPHYRIN\_AND\_CHLOROPHYLL\_METABOLISM | Details ... | 19 | -0.59 | -1.72 | 0.013 | 0.086 | 0.359 | 2569 | tags=53%, list=21%, signal=67% |
| 6 | KEGG\_ASCORBATE\_AND\_ALDARATE\_METABOLISM | Details ... | 7 | -0.74 | -1.63 | 0.021 | 0.166 | 0.637 | 1967 | tags=71%, list=16%, signal=85% |
| 7 | KEGG\_INTESTINAL\_IMMUNE\_NETWORK\_FOR\_IGA\_PRODUCTION | Details ... | 20 | -0.55 | -1.61 | 0.021 | 0.159 | 0.680 | 2746 | tags=70%, list=23%, signal=91% |
| 8 | KEGG\_LIMONENE\_AND\_PINENE\_DEGRADATION | Details ... | 7 | -0.72 | -1.57 | 0.039 | 0.198 | 0.807 | 1792 | tags=57%, list=15%, signal=67% |
| 9 | KEGG\_DRUG\_METABOLISM\_CYTOCHROME\_P450 | Details ... | 26 | -0.49 | -1.55 | 0.023 | 0.196 | 0.841 | 2550 | tags=58%, list=21%, signal=73% |
| 10 | KEGG\_GLUTATHIONE\_METABOLISM | Details ... | 34 | -0.46 | -1.55 | 0.024 | 0.177 | 0.842 | 1940 | tags=32%, list=16%, signal=38% |
| 11 | KEGG\_VALINE\_LEUCINE\_AND\_ISOLEUCINE\_DEGRADATION | Details ... | 36 | -0.45 | -1.55 | 0.021 | 0.169 | 0.853 | 1792 | tags=42%, list=15%, signal=49% |
| 12 | KEGG\_RETINOL\_METABOLISM | Details ... | 23 | -0.50 | -1.54 | 0.033 | 0.159 | 0.863 | 1068 | tags=39%, list=9%, signal=43% |
| 13 | KEGG\_BETA\_ALANINE\_METABOLISM | Details ... | 16 | -0.54 | -1.51 | 0.047 | 0.189 | 0.925 | 2270 | tags=63%, list=19%, signal=77% |
| 14 | KEGG\_AMINOACYL\_TRNA\_BIOSYNTHESIS | Details ... | 20 | -0.50 | -1.47 | 0.055 | 0.235 | 0.967 | 3334 | tags=60%, list=28%, signal=83% |
| 15 | KEGG\_PENTOSE\_AND\_GLUCURONATE\_INTERCONVERSIONS | Details ... | 11 | -0.56 | -1.41 | 0.096 | 0.319 | 0.991 | 433 | tags=36%, list=4%, signal=38% |
| 16 | KEGG\_METABOLISM\_OF\_XENOBIOTICS\_BY\_CYTOCHROME\_P450 | Details ... | 24 | -0.45 | -1.39 | 0.082 | 0.328 | 0.996 | 2550 | tags=58%, list=21%, signal=74% |
| 17 | KEGG\_CELL\_ADHESION\_MOLECULES\_CAMS | Details ... | 73 | -0.35 | -1.39 | 0.035 | 0.311 | 0.996 | 3160 | tags=42%, list=26%, signal=57% |
| 18 | KEGG\_VALINE\_LEUCINE\_AND\_ISOLEUCINE\_BIOSYNTHESIS | Details ... | 5 | -0.68 | -1.35 | 0.145 | 0.386 | 1.000 | 3386 | tags=80%, list=28%, signal=111% |
| 19 | KEGG\_SULFUR\_METABOLISM | Details ... | 6 | -0.64 | -1.33 | 0.152 | 0.412 | 1.000 | 104 | tags=33%, list=1%, signal=34% |
| 20 | KEGG\_TYROSINE\_METABOLISM | Details ... | 23 | -0.43 | -1.32 | 0.123 | 0.392 | 1.000 | 2370 | tags=43%, list=20%, signal=54% |
| 21 | KEGG\_B\_CELL\_RECEPTOR\_SIGNALING\_PATHWAY |  | 57 | -0.35 | -1.32 | 0.082 | 0.385 | 1.000 | 3347 | tags=47%, list=28%, signal=65% |
| 22 | KEGG\_STARCH\_AND\_SUCROSE\_METABOLISM |  | 26 | -0.41 | -1.30 | 0.127 | 0.413 | 1.000 | 2766 | tags=42%, list=23%, signal=55% |
| 23 | KEGG\_PEROXISOME |  | 70 | -0.33 | -1.29 | 0.086 | 0.407 | 1.000 | 1896 | tags=29%, list=16%, signal=34% |
| 24 | KEGG\_GALACTOSE\_METABOLISM |  | 23 | -0.40 | -1.23 | 0.190 | 0.535 | 1.000 | 1596 | tags=30%, list=13%, signal=35% |
| 25 | KEGG\_STEROID\_HORMONE\_BIOSYNTHESIS |  | 16 | -0.44 | -1.23 | 0.201 | 0.522 | 1.000 | 2410 | tags=56%, list=20%, signal=70% |
| 26 | KEGG\_PRIMARY\_IMMUNODEFICIENCY |  | 24 | -0.39 | -1.22 | 0.198 | 0.516 | 1.000 | 2292 | tags=42%, list=19%, signal=51% |
| 27 | KEGG\_FATTY\_ACID\_METABOLISM |  | 30 | -0.37 | -1.20 | 0.201 | 0.540 | 1.000 | 1792 | tags=37%, list=15%, signal=43% |
| 28 | KEGG\_ONE\_CARBON\_POOL\_BY\_FOLATE |  | 15 | -0.43 | -1.19 | 0.241 | 0.552 | 1.000 | 532 | tags=20%, list=4%, signal=21% |
| 29 | KEGG\_ARACHIDONIC\_ACID\_METABOLISM |  | 26 | -0.37 | -1.18 | 0.220 | 0.559 | 1.000 | 1341 | tags=27%, list=11%, signal=30% |
| 30 | KEGG\_CARDIAC\_MUSCLE\_CONTRACTION |  | 38 | -0.34 | -1.18 | 0.206 | 0.554 | 1.000 | 2839 | tags=37%, list=24%, signal=48% |
| 31 | KEGG\_BASAL\_CELL\_CARCINOMA |  | 35 | -0.34 | -1.14 | 0.250 | 0.638 | 1.000 | 2558 | tags=31%, list=21%, signal=40% |
| 32 | KEGG\_PARKINSONS\_DISEASE |  | 79 | -0.28 | -1.13 | 0.236 | 0.653 | 1.000 | 4262 | tags=48%, list=35%, signal=74% |
| 33 | KEGG\_PROPANOATE\_METABOLISM |  | 23 | -0.37 | -1.12 | 0.297 | 0.644 | 1.000 | 2270 | tags=43%, list=19%, signal=54% |
| 34 | KEGG\_VIBRIO\_CHOLERAE\_INFECTION |  | 44 | -0.31 | -1.12 | 0.267 | 0.630 | 1.000 | 1412 | tags=18%, list=12%, signal=21% |
| 35 | KEGG\_PRIMARY\_BILE\_ACID\_BIOSYNTHESIS |  | 11 | -0.44 | -1.11 | 0.332 | 0.633 | 1.000 | 2192 | tags=55%, list=18%, signal=67% |
| 36 | KEGG\_ARGININE\_AND\_PROLINE\_METABOLISM |  | 37 | -0.32 | -1.10 | 0.315 | 0.660 | 1.000 | 1874 | tags=24%, list=16%, signal=29% |
| 37 | KEGG\_HEMATOPOIETIC\_CELL\_LINEAGE |  | 47 | -0.30 | -1.09 | 0.300 | 0.661 | 1.000 | 2511 | tags=36%, list=21%, signal=46% |
| 38 | KEGG\_NEUROACTIVE\_LIGAND\_RECEPTOR\_INTERACTION |  | 121 | -0.25 | -1.08 | 0.276 | 0.666 | 1.000 | 1545 | tags=24%, list=13%, signal=27% |
| 39 | KEGG\_GLYCOSAMINOGLYCAN\_BIOSYNTHESIS\_KERATAN\_SULFATE |  | 13 | -0.41 | -1.08 | 0.371 | 0.656 | 1.000 | 2087 | tags=31%, list=17%, signal=37% |
| 40 | KEGG\_GLYCOSAMINOGLYCAN\_DEGRADATION |  | 17 | -0.38 | -1.07 | 0.372 | 0.674 | 1.000 | 3378 | tags=53%, list=28%, signal=74% |
| 41 | KEGG\_PYRUVATE\_METABOLISM |  | 30 | -0.32 | -1.05 | 0.378 | 0.710 | 1.000 | 2126 | tags=37%, list=18%, signal=44% |
| 42 | KEGG\_WNT\_SIGNALING\_PATHWAY |  | 104 | -0.25 | -1.04 | 0.366 | 0.721 | 1.000 | 1953 | tags=20%, list=16%, signal=24% |
| 43 | KEGG\_FRUCTOSE\_AND\_MANNOSE\_METABOLISM |  | 28 | -0.32 | -1.02 | 0.415 | 0.743 | 1.000 | 1676 | tags=21%, list=14%, signal=25% |
| 44 | KEGG\_RENIN\_ANGIOTENSIN\_SYSTEM |  | 13 | -0.39 | -1.02 | 0.423 | 0.735 | 1.000 | 1861 | tags=38%, list=15%, signal=45% |
| 45 | KEGG\_ACUTE\_MYELOID\_LEUKEMIA |  | 46 | -0.28 | -1.02 | 0.425 | 0.725 | 1.000 | 2796 | tags=33%, list=23%, signal=42% |
| 46 | KEGG\_NON\_SMALL\_CELL\_LUNG\_CANCER |  | 44 | -0.28 | -1.01 | 0.430 | 0.721 | 1.000 | 3087 | tags=32%, list=26%, signal=43% |
| 47 | KEGG\_CHRONIC\_MYELOID\_LEUKEMIA |  | 61 | -0.26 | -1.00 | 0.445 | 0.744 | 1.000 | 3221 | tags=36%, list=27%, signal=49% |
| 48 | KEGG\_PHENYLALANINE\_METABOLISM |  | 11 | -0.40 | -1.00 | 0.452 | 0.736 | 1.000 | 40 | tags=9%, list=0%, signal=9% |
| 49 | KEGG\_CITRATE\_CYCLE\_TCA\_CYCLE |  | 28 | -0.30 | -0.97 | 0.508 | 0.790 | 1.000 | 4007 | tags=46%, list=33%, signal=70% |
| 50 | KEGG\_ENDOMETRIAL\_CANCER |  | 44 | -0.27 | -0.97 | 0.501 | 0.778 | 1.000 | 1864 | tags=20%, list=16%, signal=24% |
| 51 | KEGG\_BASE\_EXCISION\_REPAIR |  | 25 | -0.31 | -0.97 | 0.510 | 0.765 | 1.000 | 2731 | tags=32%, list=23%, signal=41% |
| 52 | KEGG\_CALCIUM\_SIGNALING\_PATHWAY |  | 118 | -0.22 | -0.96 | 0.548 | 0.776 | 1.000 | 1514 | tags=19%, list=13%, signal=21% |
| 53 | KEGG\_PRION\_DISEASES |  | 22 | -0.31 | -0.96 | 0.514 | 0.764 | 1.000 | 2938 | tags=41%, list=24%, signal=54% |
| 54 | KEGG\_DRUG\_METABOLISM\_OTHER\_ENZYMES |  | 23 | -0.31 | -0.95 | 0.525 | 0.760 | 1.000 | 2669 | tags=48%, list=22%, signal=61% |
| 55 | KEGG\_PANTOTHENATE\_AND\_COA\_BIOSYNTHESIS |  | 12 | -0.37 | -0.95 | 0.523 | 0.753 | 1.000 | 1922 | tags=50%, list=16%, signal=59% |
| 56 | KEGG\_CYSTEINE\_AND\_METHIONINE\_METABOLISM |  | 26 | -0.29 | -0.93 | 0.575 | 0.800 | 1.000 | 821 | tags=19%, list=7%, signal=21% |
| 57 | KEGG\_PENTOSE\_PHOSPHATE\_PATHWAY |  | 19 | -0.31 | -0.91 | 0.581 | 0.826 | 1.000 | 662 | tags=16%, list=6%, signal=17% |
| 58 | KEGG\_NOTCH\_SIGNALING\_PATHWAY |  | 37 | -0.25 | -0.86 | 0.698 | 0.936 | 1.000 | 3925 | tags=51%, list=33%, signal=76% |
| 59 | KEGG\_GLYCOLYSIS\_GLUCONEOGENESIS |  | 40 | -0.24 | -0.85 | 0.716 | 0.937 | 1.000 | 2370 | tags=30%, list=20%, signal=37% |
| 60 | KEGG\_GLYCOSYLPHOSPHATIDYLINOSITOL\_GPI\_ANCHOR\_BIOSYNTHESIS |  | 19 | -0.29 | -0.84 | 0.694 | 0.945 | 1.000 | 2847 | tags=37%, list=24%, signal=48% |
| 61 | KEGG\_RIBOFLAVIN\_METABOLISM |  | 12 | -0.33 | -0.84 | 0.686 | 0.934 | 1.000 | 2188 | tags=33%, list=18%, signal=41% |
| 62 | KEGG\_PURINE\_METABOLISM |  | 112 | -0.19 | -0.83 | 0.848 | 0.928 | 1.000 | 2688 | tags=26%, list=22%, signal=33% |
| 63 | KEGG\_GLIOMA |  | 51 | -0.22 | -0.82 | 0.811 | 0.945 | 1.000 | 3221 | tags=31%, list=27%, signal=43% |
| 64 | KEGG\_AMINO\_SUGAR\_AND\_NUCLEOTIDE\_SUGAR\_METABOLISM |  | 38 | -0.23 | -0.81 | 0.796 | 0.951 | 1.000 | 1676 | tags=21%, list=14%, signal=24% |
| 65 | KEGG\_LYSINE\_DEGRADATION |  | 35 | -0.24 | -0.80 | 0.797 | 0.948 | 1.000 | 1792 | tags=17%, list=15%, signal=20% |
| 66 | KEGG\_NEUROTROPHIN\_SIGNALING\_PATHWAY |  | 99 | -0.19 | -0.79 | 0.906 | 0.941 | 1.000 | 3252 | tags=30%, list=27%, signal=41% |
| 67 | KEGG\_ERBB\_SIGNALING\_PATHWAY |  | 69 | -0.20 | -0.79 | 0.873 | 0.928 | 1.000 | 3527 | tags=35%, list=29%, signal=49% |
| 68 | KEGG\_RNA\_POLYMERASE |  | 20 | -0.26 | -0.78 | 0.788 | 0.942 | 1.000 | 3230 | tags=30%, list=27%, signal=41% |
| 69 | KEGG\_GLYCOSAMINOGLYCAN\_BIOSYNTHESIS\_CHONDROITIN\_SULFATE |  | 17 | -0.27 | -0.77 | 0.774 | 0.931 | 1.000 | 2794 | tags=29%, list=23%, signal=38% |
| 70 | KEGG\_HYPERTROPHIC\_CARDIOMYOPATHY\_HCM |  | 52 | -0.21 | -0.77 | 0.869 | 0.920 | 1.000 | 655 | tags=10%, list=5%, signal=10% |
| 71 | KEGG\_ALANINE\_ASPARTATE\_AND\_GLUTAMATE\_METABOLISM |  | 22 | -0.25 | -0.77 | 0.814 | 0.916 | 1.000 | 653 | tags=14%, list=5%, signal=14% |
| 72 | KEGG\_DILATED\_CARDIOMYOPATHY |  | 54 | -0.20 | -0.73 | 0.917 | 0.942 | 1.000 | 655 | tags=9%, list=5%, signal=10% |
| 73 | KEGG\_T\_CELL\_RECEPTOR\_SIGNALING\_PATHWAY |  | 81 | -0.18 | -0.72 | 0.955 | 0.943 | 1.000 | 3527 | tags=35%, list=29%, signal=49% |
| 74 | KEGG\_BASAL\_TRANSCRIPTION\_FACTORS |  | 26 | -0.21 | -0.66 | 0.932 | 0.980 | 1.000 | 4780 | tags=46%, list=40%, signal=76% |
| 75 | KEGG\_ASTHMA |  | 3 | -0.38 | -0.64 | 0.876 | 0.981 | 1.000 | 1243 | tags=33%, list=10%, signal=37% |
| 76 | KEGG\_DORSO\_VENTRAL\_AXIS\_FORMATION |  | 16 | -0.22 | -0.61 | 0.951 | 0.981 | 1.000 | 1891 | tags=19%, list=16%, signal=22% |
| 77 | KEGG\_HUNTINGTONS\_DISEASE |  | 122 | -0.13 | -0.57 | 1.000 | 0.981 | 1.000 | 4262 | tags=38%, list=35%, signal=58% |
Table: Gene sets enriched in phenotype **0 (3 samples) **[plain text format]****
